# A case of Schnitzler-like syndrome with basement membrane IgM deposition but without monoclonal gammopathy

**DOI:** 10.1016/j.jdcr.2024.02.024

**Published:** 2024-03-06

**Authors:** Helana Ghali, Erin M. McClure, Erin L. Foster

**Affiliations:** a University of South Florida, Morsani College of Medicine, Tampa, Florida; b Department of Dermatology, Oregon Health & Science University, Portland, Oregon

**Keywords:** chronic urticaria, IgM gammopathy, Schnitzler syndrome

## Introduction

Schnitzler syndrome is a rare autoinflammatory disorder with roughly 300 known cases worldwide. It has been described most in white males (1.5:1 male:female) with a median age of presentation of 51 years.^1^ The clinical features of the disorder are associated with increased levels of interleukin-1 (IL-1).^2^ An urticarial rash and monoclonal immunoglobulin (Ig)M or IgG gammopathy are the most distinctive features of the disorder; however, intermittent fevers, arthralgias, and leukocytosis are additional characteristics of the syndrome.^2^ Long-term risks of the disorder include progression to secondary amyloidosis and lymphoproliferation.^2^

Monoclonal gammopathy is reported in 92% to 94% of patients with symptoms of Schnitzler syndrome.^1^^,^^3^ However, there are some reports of Schnitzler syndrome presenting without a monoclonal gammopathy.^4^, ^5^, ^6^ Of the patients with a monoclonal gammopathy, 23% to 25% also have monoclonal IgM deposits in the skin along the dermoepidermal junction (DEJ) or capillary walls.^7^^,^^8^ However, the presentation of a Schnitzler-like syndrome lacking monoclonal gammopathy but featuring monoclonal IgM deposits at the DEJ has not been documented in the literature.

We present a unique case of a 47-year-old woman with Schnitzler-like syndrome without evidence of serum monoclonal gammopathy. Despite the lack of a gammopathy, this patient was also found to have IgM deposits along the DEJ on skin biopsy, along with other qualifying symptoms of Schnitzler syndrome.

## Case report

A 47-year-old woman presented with a 10-year history of pruritic rash and recurrent flares of fevers, intermittent bone pain, fatigue, and pharyngitis. For these symptoms, other providers had diagnosed her with chronic fatigue syndrome and mast cell activation syndrome but without meeting the criteria for either.

Despite taking over-the-counter antihistamines 4 times daily for more than 6 months, the rash and musculoskeletal pain remained unresponsive. The patient was also previously treated with prednisone, which partially improved the rash but was poorly tolerated. Her rash and pharyngitis improved somewhat with the avoidance of high-histamine foods. She previously took methotrexate, and is currently taking hydroxychloroquine, for her unrelated history of palindromic rheumatism; these did not provide a clear benefit.

**Fig 1:**
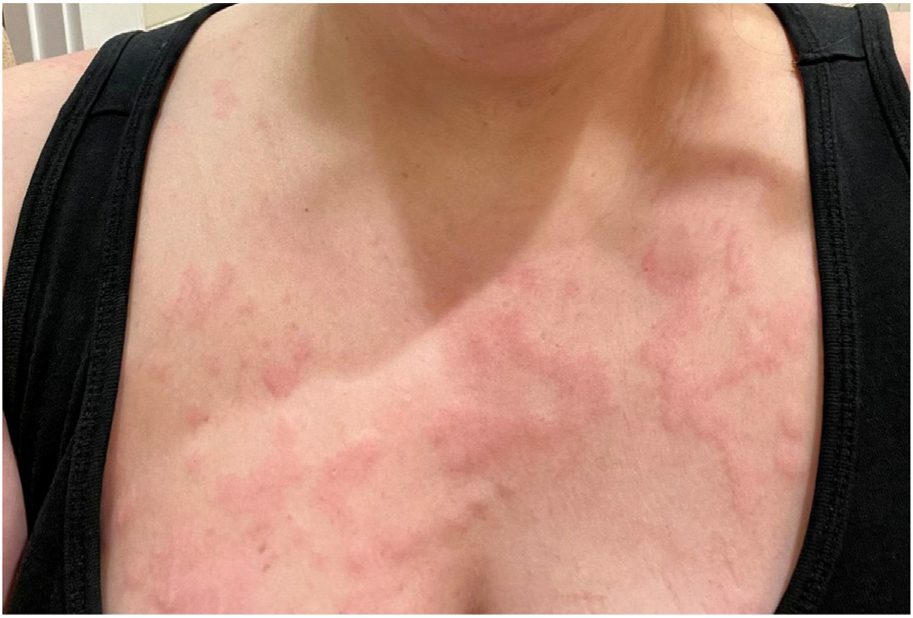
Superior chest with smooth pink edematous plaques and papules with firm infiltrative nodular components.

**Fig 2:**
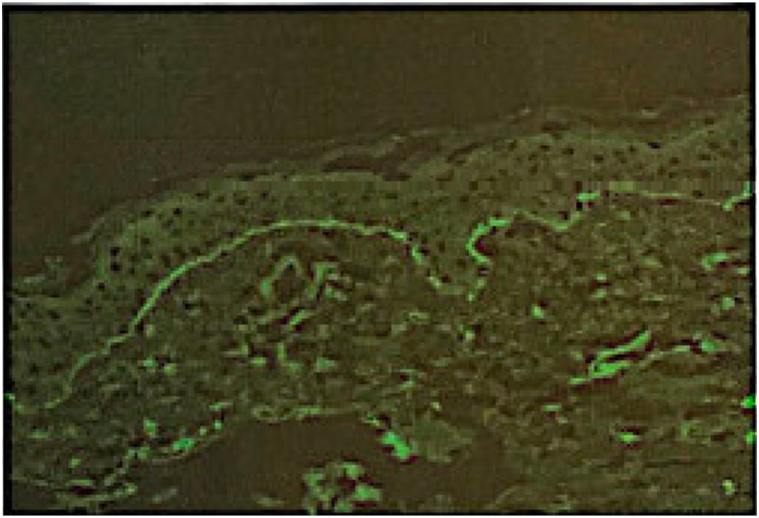
Direct immunofluorescence with 2+ linear deposition of IgM along the basement membrane. *IgM*, Immunoglobulin M.

A skin exam revealed smooth pink edematous plaques and papules located on the trunk and extremities, some with firm infiltrated nodular components (Fig 1) and scattered bright pink erosions on the hips. Biopsy findings of the left upper arm on hematoxylin and eosin stain showed a sparse perivascular and interstitial infiltrate of lymphocytes, eosinophils, and some neutrophils. Direct immunofluorescence (DIF) showed a linear deposition of IgM along the basement membrane (Fig 2). She had no palpable lymphadenopathy.

Laboratory workup revealed elevated erythrocyte sedimentation rate, C-reactive protein, and C3. Kidney, thyroid, and liver function tests were normal, and antinuclear antibody was negative. Serum IgG, IgM, and IgA levels were normal. A bone marrow biopsy showed no evidence of lymphoplasmacytic lymphoma. Next generation sequencing and fluorescence in situ hybridization were negative for myeloid differentiation primary response 88 mutation. Bone marrow aspirate, clot section, core biopsy, and peripheral blood were morphologically and immunophenotypically unremarkable. Skeletal survey revealed normal bone mineralization with multilevel degenerative changes throughout the thoracolumbar spine, and mild bilateral tibiofemoral compartment joint space narrowing with lateral osteophyte formation. Serum free light chain and serum protein electrophoresis were within normal limits, and urine immunofixation was negative for monoclonal free light chains.

The patient was bridged with dapsone for 1 week until she was started on anakinra, and her symptoms dramatically improved within 3 weeks. Now 4 months into treatment, she reports a complete resolution of her symptoms.

## Discussion

The presence of monoclonal IgM is a mandatory factor for diagnosing Schnitzler syndrome, used in 2 sets of validated criteria for this condition.^2^ The Lipsker criteria includes urticarial rash and monoclonal IgM, with 2 or more of fever, arthralgia, bone pain, palpable lymphadenopathy, liver or spleen enlargement, elevated erythrocyte sedimentation rate, leukocytosis, or abnormal bone scan.^2^ The Strasbourg criteria distinguishes patients into definite or probable diagnoses with the major criteria being urticarial rash and monoclonal gammopathy of IgG or IgM, and minor criteria of recurrent fever, bone remodeling, dermal neutrophilic infiltration, and leukocytosis.^2^

The top differential diagnoses of this case include a combination of chronic urticaria and the patient’s established diagnosis of palindromic rheumatism, or a cryopyrin-associated periodic syndrome (familial cold autoinflammatory syndrome or Muckle-Wells syndrome) as these conditions would explain the rash and systemic symptoms. However, in most biopsy reports of chronic urticaria that included DIF, IgM immune deposits were not detected at the DEJ.^9^^,^^10^ Furthermore, the patient did not respond to therapy with high-dose antihistamines or antiinflammatories, which should be effective in chronic urticaria and palindromic rheumatism, respectively. The patient did not have a relevant family history, sensitivity to cold, sensorineural deafness, or other indicators of systemic inflammation which would be expected in a cryopyrin-associated periodic syndrome. Hyper-IgD syndrome was also considered, as this too can present with an urticarial rash, fevers, and arthralgias, but the patient did not have lymphadenopathy or abdominal pain. Other diagnoses that were considered include adult-onset Still disease and rheumatoid arthritis; however, the patient’s urticarial rash was not consistent with these conditions.

The constellation of clinical symptoms, laboratory findings, DIF results, and quality of life improvement following IL-1 inhibition favor a diagnosis of Schnitzler-like syndrome, despite the absence of a monoclonal gammopathy. Notably, her serum IgM levels were normal even in the presence of IgM deposition at the basement membrane, highlighting the need for a nuanced understanding of diagnostic criteria beyond monoclonal gammopathy.

While most reports of Schnitzler syndrome include a monoclonal gammopathy (Table I), there are reported cases of Schnitzler syndrome with absent or delayed gammopathy (Table II). Therefore, a monoclonal gammopathy may develop in this patient in the future. Based on the findings of this case, we suggest consideration of broadening the spectrum of Schnitzler-like disease to permit qualifying cases without a serum monoclonal gammopathy. This could enhance the outcomes of similar cases that do not respond to high-dose antihistamines with the use of IL-1 inhibition. Additionally, DIF may be considered to aid in the diagnosis of suspected Schnitzler-like conditions; however, further research is needed to better understand the involvement of IgM deposition in the pathomechanism of autoinflammatory disorders such as Schnitzler syndrome.

**Table I:** Number of Schnitzler syndrome cases in the literature with monoclonal gammopathy at time of disease presentation, delayed monoclonal gammopathy, or absent monoclonal gammopathy

Monoclonal gammopathy	Delayed monoclonal gammopathy	No monoclonal gammopathy
260+	3	10

**Table II:** Previous case reports of Schnitzler syndrome without monoclonal gammopathy with response to IL-1 blockade

Author, y	Age (y)	Sex	Related systemic symptoms	Serum IgM levels	H&E	IL-1 blockade treatment response (+/−)
Husak R, 2000	57	M	Severe recurrent urticaria, joint and bone pain, and intermittent fever	6.5 g/L	Perivascular lymphomononuclear cell and granulocyte infiltrate in the dermis and mild leukocytoclasis	N/A
Varella TCN, 2005	36	M	Severe chronic nonpruritic urticaria, arthralgia, bone pain, and intermittent high-grade fever	3.46 g/L	Neutrophilic urticaria	N/A
Treudler R, 2007	58	F	Erythematous macular and partly urticarial rash, anemia, fatigue, and arthralgias	Not reported	Interstitial perivascular mononuclear and polymorphonuclear cell infiltrate and superficial dermal edema	+
Chu CQ, 2010	54	F	Chronic urticaria and diffuse joint pain, fatigue, and night sweats	Within normal limits	Neutrophilic infiltration	+
Gladue HS, 2014	44	F	Chronic urticaria, intermittent fever, arthralgias, and recurrent angioedema	Hypogamma-globulinemia	Perivascular lymphocytic infiltrate with interstitial eosinophils and neutrophils	+
Urbanski M, 2016	62	M	Diffuse urticaria, fatigue, nonproductive cough, arthralgias, and intermittent fever	Within normal limits; not reported	Neutrophilic urticaria and absence of small capillary fibrinoid necrosis and no red blood cell extravasation	+
Ahn MJ, 2018	69	M	Fever, chronic urticaria, arthralgia, and myalgia	1.54 g/L	Neutrophilic and eosinophilic inflammation, no evidence of vasculitis	+
Fujita Y, 2021	43	F	Recurrent febrile attacks, and arthralgias accompanied by urticarial eruption	Not reported	Neutrophilic, lymphocytic, and eosinophilic perivascular dermal infiltration	+
Bixio R, 2021	21	F	Recurrent episodes of nonpruritic urticaria, cutaneous angioedema, arthritis, arthralgias, and fever	2.75 g/L	Leukocytoclastic neutrophilic infiltration, minimal dermal edema	+
WesselmannAS, 2023	75	M	Recurrent nonpruritic, urticarial plaques without arthralgia or fever	Not reported	Perivascular and interstitial neutrophilic infiltrate, neutrophilic epitheliotropism	+
This report, 2023	47	F	Recurrent flares of fevers, arthralgias, fatigue, and urticarial rash	Within normal limits	Perivascular and interstitial infiltrate of lymphocytes, eosinophils, and neutrophils	+

Excluded cases with delayed presentations of monoclonal gammopathy.

## Conflicts of interest

None disclosed.
